# The Psychology of Coronavirus Behavioral Health Mindset, Vaccination Receptivity, Customer Orientation and Community Public Service

**DOI:** 10.3389/fpsyg.2022.837365

**Published:** 2022-04-14

**Authors:** Michael R. Cunningham, Perri B. Druen, M. Cynthia Logsdon, Brian W. Dreschler, Anita P. Barbee, Ruth L. Carrico, Steven W. Billings, John W. Jones

**Affiliations:** ^1^Department of Communication, University of Louisville, Louisville, KY, United States; ^2^Department of Psychology, York College, York, PA, United States; ^3^School of Nursing, University of Louisville, Louisville, KY, United States; ^4^FifthTheory, LLC., Chicago, IL, United States; ^5^Kent School of Social Work, University of Louisville, Louisville, KY, United States; ^6^Norton Healthcare, Louisville, KY, United States

**Keywords:** COVID-19, coronavirus, scale validation, health behavior, facemask wearing, social distancing, anti-vaccination sentiments

## Abstract

Three studies were conducted to explore the psychological determinants of COVID-deterrent behaviors. In Study 1, using data collected and analyzed both before and after the release of COVID-19 vaccines, mask-wearing, other preventative behaviors like social distancing, and vaccination intentions were positively related to assessments of the Coronavirus Behavioral Health Mindset (CVBHM); belief in the credibility of science; progressive political orientation; less use of repressive and more use of sensitization coping; and the attribution of COVID-19 safety to effort rather than ability, powerful forces, fate, or luck. In Study 2, favorable COVID-19 vaccination intentions were related to greater willingness to work, lower emotional distress, and greater customer experience mindset. Study 3 examined the personality and motives of individuals who volunteered to help deliver COVID-19 inoculations to the local community. The vaccine-giving volunteers, especially those with prosocial motives, had high CVBHM scores, belief in the credibility of science, low use of repressive coping, greater attribution of COVID-19 protection to effort, low likelihood of voting conservative, were older, and had more education than others. The majority of public health volunteers expressed prosocial motives to help people or join a cause (60.7%), but many (39.3%) expressed the personal motives of getting the COVID-19 vaccination for themselves, conveying a public image of compassion, or structuring time. Based on the three research studies, a COVID-19 Mindset Hierarchy model is proposed to integrate the results.

## Introduction

The SARS-CoV-2 pandemic, commonly known as COVID-19, devastated countries around the world. In this context, the pandemic provides a window into the relation between individual differences in cognitions, emotions, and behaviors on the one hand, and health-related decision-making on the other. Early in the pandemic, the United States Centers for Disease Control and Prevention (CDC) released guidelines to reduce the spread of the COVID-19 virus, including use of respiratory protection such as face masks, social distancing, and avoidance of settings in which large numbers of people were in close proximity (CDC, 2020a,b, c, d, e) but not everyone chose to heed the call. In March of 2020, our research team developed a new instrument called the Coronavirus Behavioral Health Mindset assessment (CVBHM). The instrument was designed to protect consumers and workers by assessing biosafety risk through acceptance of responsibility for protection of oneself and others, social distancing, and adhering to prevention measures (Dreschler et al., 2020; Cunningham et al., 2021).

The CVBHM is grounded in decades of theory-based research on safety locus of control (LOC). Safety LOC theory posits that individuals who possess an internal LOC take personal responsibility for safety whereas individuals with an external LOC for safety see self-protection as an excessively difficult challenge and attribute mishaps to uncontrollable forces and bad luck. Such differences in attitude lead people with an internal LOC for safety to engage in greater vigilance and carefulness and have fewer accidents than those with an external LOC for safety (Jones and Wuebker, 1985, 1993; Wuebker, 1986; Ng et al., 2006). Using a brief safety LOC measure, Cunningham (2002) differentiated between food retail employees and utility workers who received a safe versus an unsafe supervisor rating, as well as between those who did and did not commit Department of Motor Vehicle offenses. An accident LOC scale also successfully separated public park employees who were involved in collisions and wrecks from those who were not (Janicak, 1996). Internal safety LOC in young farm workers was associated with greater likelihood of acknowledging mistakes that would cause accidents at work (Cigularov et al., 2009). Aviators who reported lower internal safety LOC had more recent accidents (Hunter and Stewart, 2012) and more hazardous events such as close calls (Joseph et al., 2013), presumably because of the effect of internality on the belief that accidents pose greater risks (You et al., 2013). In the construction industry, individuals with an external safety LOC had more actions deemed unsafe at work (Kuo and Tsaur, 2004). Finally, those with a more internal safety LOC were less likely to enact improper or criminal acts in nuclear energy facility management (Cunningham and Jones, 2013).

Besides incorporating safety LOC, the CVBHM leveraged insights from the theory of health LOC (Wallston et al., 1978; Weiss and Larsen, 1990). An internal health LOC is associated with health promoting behaviors overall (Wallston, 1992; Cheng et al., 2016), particularly the self-mastery part of control (Marshall, 1991), and especially for those seeking to protect their own health (Norman et al., 1998). Weiss and Larsen (1990) found that individuals with an internal health LOC had higher scores on health-oriented behavior measures, such as getting a flu shot, but only when the participants were focused on the value of their health. People with an external health LOC were particularly susceptible to the negative influence of social contacts who did not engage in health promoting behavior (Abella and Heslin, 1984). Most recently, Pitel and Mikušková (2021) studied the health behaviors of 448 students attending Slovak universities. They found that an internal health LOC was positively correlated with physical exercise and fruit and vegetable consumption, and negatively correlated with soft drink consumption and with irrational health beliefs, including a belief in powerful others and chance.

Our 30-item CVBHM assessment, designed to reflect both the safety and health LOC theories and findings, was evaluated in two surveys of 1,455 respondents (Time 1) and 431 respondents (Time 2) using data gathered between March 2020 and July 2020. The CVBHM was found to have strong internal consistency (T1 α = 0.90, T2 α = 0.88) and test–retest reliability (*r* = 0.84). More importantly, the CVBHM demonstrated high predictive validity with the criterion of wearing a facemask (T1 β = 0.64, T2 β = 0.60). The CVBHM was a stronger predictor of COVID-19 safety behavior than other individual differences, although demographics, employment status, personality, and vocational interest were related. Lower CVBHM scores were reported by participants who described themselves as conservative or libertarian, rather than progressive (*r* = –0.30) and as male rather than female (*r* = –0.17). Higher CVBHM scores were found for older individuals than younger individuals (*r* = 0.28) and for those participants with more education as opposed to less (*r* = 0.18).

On the Five-Factor Model (FFM) of personality, high scorers on the CVBHM were more agreeable (*r* = 0.23), more conscientious (*r* = 0.15) and had more openness to new ideas and experiences (*r* = 0.15) than their lower scoring counterparts. Those results are consistent with prior studies on the FFM, which found that conscientiousness and agreeableness were associated with lower rates of unsafe behavior (Clarke and Robertson, 2005; Beus et al., 2015). On the Holland vocational interest typology, individuals who were interested in realistic careers (*r* = –0.18), such as airplane piloting and bricklaying scored lower on the CVBHM, whereas those who were interested in social careers, like working with children (*r* = 0.17), or artistic careers such as music (*r* = 0.13) scored higher on the CVBHM.

As will be described below, our research team initiated further investigations of the CVBHM, testing for relationships with other individual difference variables, and with additional COVID-19 related behaviors and coping variables. While we were gathering our data, we noted other findings providing insights on the relation of attitudes and personality to COVID-related health behaviors. Just as the CVBHM focuses on the cognitive determinants of health-related behaviors, especially internal versus external LOC on COVID-19 prevention practices, other studies also used the LOC construct to predict coping with COVID. Barron-Millar et al. (2021) surveyed 234 members of an Indian community during the first week of COVID-19 lockdown. Higher scores on both internal and external LOC measures were associated with anxiety, but they also were associated with different coping strategies. An internal LOC was associated with behavioral coping (reading, resting, learning something new, being with family) and coping through social media (increased use of social media, using technology to connect with my loved ones, reading, and enjoying humorous messages and sharing with others). An external LOC was associated with “mindfulness” coping (practicing mediation, starting exercises and yoga at home, resisting thoughts of illness).

Studies that were conducted in a diverse set of countries from Japan and Qatar to Slovenia and Brazil examining the FFM dimensions and COVID-prevention produced mixed results. A study in Qatar distributed a questionnaire to the mobile phone of individuals connected to the author’s social networks (*n* = 418, Abdelrahman, 2020). Significant positive correlations were found between both conscientiousness (*r* = 0.27) and neuroticism (*r* = 0.18) with social distancing, likely due to responsibility and fear, as well as a significant negative correlation between agreeableness with social distancing (*r* = –0.11), perhaps because such people craved closeness more than safety. Working in Brazil, Carvalho et al. (2020) conducted an online survey of 715 people and found small but significant relations between extraversion (*r* = 0.09) and conscientiousness (*r* = 0.06) with a measure combining social distancing and handwashing. Aschwanden et al. (2021) used a third party to recruit 2,066 U.S. participants to complete an online study. They found significant relations between mask-buying and extraversion (*r* = 0.19) but no significant associations with neuroticism (*r* = 0.00), openness (*r* = –0.01) or agreeableness (*r* = –0.09); while conscientiousness was negatively related (*r* = –20), perhaps because this group was vigilantly taking other precautions. By contrast, a study in Slovenia with 963 participants gathered through social media found only conscientiousness to be significantly correlated with mask-wearing, with no relation found for the other four FFM dimensions (Turk et al., 2021). Lastly, data on personality traits and adherence to COVID-19 transmission mitigation guidelines were gathered from 8,548 Japanese citizens by survey in March 2020 (Nofal et al., 2020). Agreeableness, conscientiousness, and openness to experience was positively related to the tendency of people to comply with safety guidelines, whereas extraversion was negatively related to the tendency of people to comply with transmission mitigation behavioral guidelines, likely due to the relation of extraversion to self-assertion and dominance.

An investigator in the United Kingdom had access to a nationally representative longitudinal study (*n* = 5,178) that began in 1958 and continues through the present. In a 2020 wave of data collection, information was obtained related to COVID-19 health status. The 2020 analysis examined the effect of Big Five personality traits on compliance with social distancing requirements and contraction of COVID-19 (Kanazawa, 2021). Conscientious individuals and females were more likely to maintain social distance and were less likely to contract COVID-19 than others. They also found that agreeable individuals were more likely to say that they were complying with social distancing requirements yet were more likely to contract COVID-19, raising questions about their veracity. Also, more likely to contract COVID-19 were neurotic and open individuals, who were no less likely to comply with precautionary guidelines than more stable and closed people. Iqbal (2021) also examined the relation between the FFM and the health protection behaviors carried out during the COVID-19 pandemic. Young adults (*n* = 680; 74% women, mean age of 22) found that both conscientiousness (C) and openness (O) to experience were associated with a higher frequency of elbow covering when sneezing (C, β = 0.11; O, β = 0.09) and practicing social distancing (C, β = 0.14; O, β = 0.10) suggested by the government, and in carrying out healthy activities during confinement.

Other studies examined the relation of negative personality dimensions to behaviors in response to COVID. Ścigała et al. (2021) conducted a meta-analysis of papers produced in 2020 and 2021 on the relation between interpersonally aversive personality traits like Machiavellianism, narcissism, psychopathy, and (low) honesty-humility as predictors and such criteria as negative affect, perception of safety guidelines, health behaviors, and prosocial behaviors. Across 34 studies with a combined sample size of 26,780 participants, they found that people with higher scores in aversive personality traits were less likely to: perceive those guidelines and restrictions to curb the spread of the virus were protective (*r* = –0.11), engage in health behaviors related to COVID-19 (*r* = –0.16), or engage in non-health related prosocial behavior related to COVID-19 (*r* = –0.14). In a related study that was not included in the meta-analysis, Hughes and Machan (2021) found that psychopathy, Machiavellianism, and collective narcissism were linked to belief in COVID-19 conspiracy theories (“There is no real evidence that COVID-19 exists.”; “COVID-19 was created in a laboratory.”).

Consistent with our prior study (Cunningham et al., 2021) and many news media polls, non-compliance with CDC recommendations with respect to COVID-19 have been linked both to ethnic group membership and political attitudes. Ethnic minorities in the U.S. have a long history of health care inequities, including unethical medical treatment in cases such as the 1932–1972 Tuskegee Syphilis Study (Thomas and Quinn, 1991). Such experiences can cause ethnic minorities to be wary of health care advice from conventional channels like the CDC (Alcendor, 2020).

Our prior investigation found that a second group, individuals with conservative political sentiments who tend to vote Republican, also displayed less safety-conscious responses to COVID-19 (Cunningham et al., 2021). During the period of the study, Republican U.S. President Donald Trump consistently downplayed the pandemic, denigrated efforts to prevent the spread of the disease and concealed the seriousness of his own encounter with the illness, which required substantial medical intervention. As a consequence, many Trump supporters adopted COVID-minimizing attitudes. Calvillo et al. (2020) reported two studies finding that political conservatism was associated with perceiving less personal vulnerability to the virus and the virus’s severity as lower, and stronger endorsement of the beliefs that the media had exaggerated the virus’s impact and that the spread of the virus was a conspiracy. Further, in four studies (total *n* = 4,441) conducted in the U.S., conservatism predicted less accurate discernment between real and fake COVID-19 headlines and fewer accurate responses to COVID-19 knowledge questions (Ruisch et al., 2021). These researchers examined the factors that contributed to ideological differences in pandemic responses and found that specific attitudes, such as trust in science and trust in President Donald Trump had a greater impact than general personality factors such as empathic concern, disgust sensitivity, and conspiratorial ideation.

As previously mentioned, the CVBHM is a targeted attitude assessment that has already demonstrated predictive relations with COVID-19 abatement behaviors. Study 1 in the current series was designed to examine the subfactors of internal and external LOC and test them against a broader range of criteria than in our earlier report. Weiner’s (1986) classic studies of the attribution of success and failure, which were based on the theoretical work of Heider (1958), suggested that people respond to challenges involving the prospect of success or failure in terms of four characteristic categories of attributions. The dimensions of internal versus external cause were subdivided as a function of whether the cause is stable and unchangeable versus unstable and variable. Personal ability is an Internal-Stable cause whereas personal effort is an Internal-Unstable cause. By contrast, powerful others and task difficulty are External-Stable causes, while luck and fate are External-Unstable causes. We predicted that attributing COVID-19 safety to effort would be associated with a higher CVBHM score and greater use of self-protective safety measures. By contrast, attributing COVID-19 safety to personal ability, powerful others, task difficulty, luck, or fate will be associated with a lower CVBHM score and less use of self-protective safety measures.

Study 1 also focused on the theoretically based coping styles of repression and sensitization (Byrne, 1961), which seem relevant to one’s response to the anxiety-provoking threat of COVID-19. Individuals who employ repression coping strategies tend to avoid thoughts and stimuli that provoke anxiety, generally by ignoring, minimizing, or denying the threat (Krohne and Rogner, 1982). Individuals disposed to sensitization coping strategies tend to approach the threat by vigilantly searching for information about it, including obsessing to the point of worry and distress. Although some individuals may be inclined either to repression or sensitization, the two dimensions can operate independently. Decades of research have found repression and sensitization to be related to the processing of threat-related information pertaining both to health (Witte and Morrison, 2000; Hastall and Wagner, 2017) and crises (Kim et al., 2014). Individuals with sensitizing tendencies, and with an external LOC, tend to have greater death anxiety than others (Tolor and Rexnikoff, 1967). Perhaps due to the adverse impact of anxiety on the immune system, sensitizers also tended to be more prone to actual illness than others (Byrne et al., 1968; Gayton et al., 1976). The former study also found that male student sensitizers made more visits to the university health center than male repressors, but there was no relation for females.

While this study was underway, in December of 2020, the U.S. Food and Drug Administration (FDA) made available the first COVID-19 vaccine through Emergency Use Authorization. As soon as we could obtain IRB approval, we added content valid questions to assess intention to receive a COVID-19 vaccination (Vx). We explored three related research questions: (a) whether a broad range of COVID-19 abatement behaviors would be predicted by the CVBHM, attributions, attitudes and coping strategies; (b) whether the CVBHM would predict intention to receive a COVID-19 Vx; and (c) whether the introduction of the COVID-19 Vx reduced participants’ commitment to engage in other COVID-19 abatement measures.

## Study 1: Method

### Participants

The Study 1 sample responded between September 18, 2020 and May 23, 2021 to solicitations for student, faculty, and staff participation in this survey at a midwestern university and a small mid-Atlantic college. Some of the students received extra credit in their classes for participation. The research was approved by the Institutional Review Boards at both institutions (IRB #20.0300 at UofL and IRB# 20SP014 at York College). On December 11, 2020, the U.S. Food and Drug Administration issued an Emergency Use Authorization for the Pfizer-BioNTech vaccine for the prevention of coronavirus disease (Food and Drug Administration, 1973). The Pre-Vx sample (*n* = 414) participated prior to December 11 and the Mid-Vx sample (*n* = 410) participated on or after that date, with the vast majority of the latter group contributing in February, March, and April of 2021 (*n* = 385).

Table 1 presents the demographic means and standard deviations. The subgroups did not significantly differ in gender: overall, 30.5% of participants identified as male, 67.9% identified as female, and 1.6% reported “other” or declined to answer. Participants were generally young, with the Pre-Vx sample younger than the Mid-Vx sample. In the Pre-Vx sample, 88.5% were in the 18–24-year-old category, compared to 67.0% in the Mid-Vx sample.

**TABLE 1:** Demographic differences between Pre-Vx and Mid-Vx samples (Study 1).

		Pre-Vx	Mid-Vx	*t*	*p*
Variables	*N*	414	410		
Sex	Mean	1.69	1.74	–1.34	0.18
	Standard deviation	0.49	0.48		
Age	Mean	2.33	2.79	–5.18	0.000
	Standard deviation	1.02	1.44		
Ethnicity	Mean	1.22	1.16	2.12	0.03
	Standard deviation	0.41	0.36		
Education	Mean	2.99	3.98	–7.60	0.000
	Standard deviation	1.46	2.10		
Employed	Mean	2.99	3.98	–5.24	0.000
	Standard deviation	1.46	2.1		

*Sex Male = 1; Other = 2; Female = 3. Age 1 = 17 years or younger; 2 = 18-24; 3 = 25–34; 4 = 35–44; 5 = 45–54; 6 = 55–64; 7 = 65–74; 8 = 75 and older. Ethnicity White = 1, Minority = 2. Education 1 ≤ HS or GED; 2 = HS or GED; 3 = 1–2 years college; 4 = Assoc. degree; 5 = 3–4 years college; 6 = Bach. degree.; 7 = some post-grad.; 8 = Masters; 9 = Doctoral or prof. degree. Employed 1 = unemployed; 2 = part-time or student; 3 = dislocated due to COVID; 4 = retired or full disability; 5 = full-time employed.*

The two samples varied somewhat in their educational attainment. In the Pre-Vx sample, 49.8% reported having a high school diploma, G.E.D. or less, 30.0% had 1–2 years of college, 4.6% had 4-year college degrees, and 2.2% had a Master’s or Doctorate degree, with the remaining having other levels of education. The Mid-Vx sample had received a bit more education: 25.3% reported having a high school diploma, G.E.D. or less, 35.5% had 1–2 years of college, 7.3% had 4-year college degrees, and 11.5% had a Master’s or Doctorate degree, with the remaining having other attainments.

The Pre-Vx sample was 84.3% white and 15.7% minority, including 6.9% black or African American. The Mid-Vx sample had less diversity and was 95.4% white and 4.6% minority, including 2.4% black or African American. The groups did not differ in the proportion reporting themselves to be Hispanic; overall that was 9.8%.

Less than half of the sample was gainfully employed. Among the Pre-Vx sample, 11.5% worked full-time and 30.3% had part-time employment, whereas 43.0% were full-time students. Economic dislocations due to COVID-19 affected 5.9% of the Pre-Vx sample. The small number remaining participants were in other employment categories, such as disabled or retired. Among the Mid-Vx sample, 24.8% worked full-time and 24.3% had part-time employment, whereas 32.9% were full-time students. Economic dislocations due to COVID-19 affected 3.2% of the Mid-Vx sample, with the rest in other categories.

We also asked two questions about political sentiments: “What is your political orientation: (Republican, Libertarian, Independent, Democratic, Green Party, Other). Just 1.5% indicated Libertarian so they were combined with Republicans, and 0.4% reported Green so they were combined with Democrats. A total of 16.0% reported Independent, and 9.5% indicated other so they were grouped together, creating three categories. Respondents also were asked “In the 2020 Presidential election, you expect to vote (or you voted) for” [Republican, Independent, Democratic, Not eligible to vote, Do not plan to vote (did not vote)]. Just 1.3% were not eligible to vote so they were dropped. The 9.2% who reported that they did not vote were grouped with the Independents, to maintain statistical power. The orientation and voting variables correlated *r* = 0.78, so we focused on the voting variable. The political affiliation of the Pre-Vx and Mid-Vx samples groups were quite similar with 27.9 and 27.2% voting Republican or Libertarian, 20.7 and 21.1% voting Independent or not voting, and 51.4 and 51.7% voting Democratic or Green party, respectively. The analyses used a 3-point scale with Democratic = 1, Independent/unaffiliated/non-participating = 2 and Republican = 3. The Republican/Libertarian group is referred to as “voting conservative” in the analyses below.

Because of the use of a volunteer academic convenience sample, the demographics of the respondents varied from that of the U.S. population. Although that fact affects descriptive statistics like means and percentages, it generally has minimal impact on correlations and contrasts. Following the recommendation of Rosenthal (1984), the correlation statistic “*r*” is presented as a common metric to indicate the strength of the linear relationship in all analyses, although other informative statistics also are presented. The square of the correlation coefficient indicates the variance accounted for in an analysis.

### Measures

#### Coronavirus Behavioral Health Mindset Assessment

Items were created to reflect a sense of personal responsibility and internal LOC, as well as behavioral intention to incorporate and comply with CDC guidelines for social distancing and disease prevention (Cunningham et al., 2020). The Ajzen–Fishbein theory of attitude-behavior congruence (Ajzen and Timko, 1986; Fishbein and Ajzen, 2011) was relied on to ensure that the COVID-19-related attitudes assessed with the CVBHM were tightly aligned with the CDC’s prescribed behaviors. Personal responsibility was reflected in items such as “I listen to the news to learn how to avoid getting coronavirus.”, and the reverse-scored item: “I think people are exaggerating the seriousness of COVID-19 health risk.” Social distancing was expressed in items such as “I try to stay six feet or more from other people to avoid contracting COVID-19.”, and the reverse-scored item: “I regularly attend social gatherings since I do not fear getting infected.” Other biosafety concepts were conveyed by items such as “I would rather telecommute than go to an office to work to avoid the virus.”, “I think it is very smart to close schools and workplaces to avoid the spread of coronavirus.”, “I wash my hands regularly during this coronavirus scare.”, and the reverse-scored item “I still shake hands with people even if I do not know if they have the virus.” Although multiple constructs were incorporated in the CVBHM, the intention was to employ it as a unidimensional scale. The CVBHM may be obtained from FifthTheory LLC, Chicago, IL at no charge.

#### Credibility of Science

In addition to the CVBHM, the Credibility of Science scale (Hartman et al., 2017) was used. This scale consists of six items assessing trust versus distrust in science (“People trust scientists a lot more than they should.” and “A lot of scientific theories are dead wrong.”).

#### COVID-19 Attributions

Items were created specifically for this study to assess attributions about COVID-19 safety and prevention in the four Weiner (1986) categories:

**Internal-Stable Attributions for COVID-19 Prevention (Personal Ability)** focus on relatively unchanging personal qualities that might lead to protection against the illness (seven items, α = 0.84). Such personal qualities include having good health, young age, relatives who lived for a long time, a positive attitude, living in an area where the infection rate is low, and being smart. Example items: “I have common sense and that tells me more about the coronavirus than any group of scientists.” and “I’m healthy enough so that if I get the coronavirus, I’m certain it will be a mild case.”

**Internal-Unstable Attributions for COVID-19 Prevention (Effort)** focus on intentional and changeable behavioral efforts to protect oneself against the illness (six items, α = 0.70). Such actions include following a ritual of mask-wearing and hand-sanitizing, following experts’ rules, engaging in healthy behavior, paying attention to the news, trying to understand from the experts, reading about all new findings. Example items: “I engage in healthy behaviors so that I can resist the coronavirus.” and “I pay attention to the news, so I learn what will prevent me from getting the coronavirus.”

**External-Stable Attributions for COVID-19 Prevention** (**Powerful Forces and Task Difficulty**) focus on relatively constant extrinsic factors, such as powerful politicians who offer protection, origin stories that moderate the seriousness or difficulty of avoiding the pandemic, or social forces that deter preventative measures (six items, α = 0.74). These factors include trusting that political leaders will keep people safe, believing that the pandemic is a hoax and that the virus was released by America’s enemies, believing that people should keep the economy going at the risk of their health and that people cannot avoid friends who want to socialize, and reports that they give in to friends who make fun of mask-wearing. Example items: “I trust my political leaders, so I am not very concerned about the coronavirus.” and “Close friends and loved ones who don’t live with me insist on socializing with me, and I cannot say no.”

**External-Unstable Attributions for COVID-19 Prevention** (**Fate and Luck**) focus on variable and unpredictable extrinsic factors such as fate and luck (six items, α = 0.85). The factors include the belief that the respondent will die when it is their time, that their fate in the hands of a higher power, that staying healthy is a matter of fate, not getting the coronavirus is a matter of luck, and the coronavirus will vanish quickly. Example items: “I will die when it is my time, so I do not need to take special precautions to avoid the coronavirus.” and “Like the yearly flu, the coronavirus will soon go away.”

#### COVID-19 Coping Responses, Precautions, and Vaccine Receptivity

**COVID Repression** (five items, α = 0.82) focused on denial and minimization. Items included the respondents reporting that they ignore the coronavirus, do not think about the coronavirus, distract themselves from coronavirus news, ignore scary news, and resent officials who insist on mask-wearing. Example items: “I don’t want my daily routine to be turned upside down, so I ignore the coronavirus.” and “News broadcasters get high ratings by scaring people, so I ignore their coronavirus reports.”

**COVID Sensitization** (six items, α = 0.68) focused on hypervigilance about the pandemic, with accompanying distress. These items included respondents reporting that they think about the pandemic when it will not have a useful outcome, that they cannot stay away from all the breaking news about the pandemic, being irritated about how other people are handling the pandemic, that stress from the pandemic is taking a toll, that they are lonely since the start of the pandemic, and they are consuming more alcohol and drugs since the start of the pandemic. Example items: “I can’t stay away from all of the breaking news about the ways the coronavirus is spread.” and “I complain a lot to others about how people are handling the pandemic.”

**COVID-19 Precautions** (eight items, α = 0.87). Throughout the COVID-19 pandemic, wearing a face mask was a primary recommended safety procedure and a flashpoint of political controversy. Besides mask-wearing, eight items measured additional recommended safety procedures, including using sterilizing wipes on newly purchased items before using them, and disinfecting one’s living space each day. Further precautions included not touching one’s face unless one’s hands had been recently washed, not eating indoors in restaurants, not going into specific shops where the employees did not seem careful about the coronavirus, not participating in crowded venues like concerts and ball games because people did not maintain a six-foot social distance, declining to participate in face-to-face educational activities for the same reason, and refusing to meet with friends and family who were not careful about the coronavirus.

**COVID-19 Vaccination Receptivity** (17 items, α = 0.93). After the FDA authorized the first COVID-19 vaccine, IRB approval was obtained in January 2021 to ask additional questions. Participants in the Mid-Vx sample were asked to respond to 17 items measuring COVID-19 vaccine receptivity. These items included confidence that the vaccine would be effective in preventing illness, believing that it should help control the spread of the infectious disease, and support for requiring all workers to get the COVID-19 vaccination. Reverse-scored items included believing that a positive attitude will protect one against COVID-19 better than any vaccine, worrying that the COVID-19 vaccine will have too many adverse side effects, and believing that no person or organization should be able to require someone to take the COVID-19 vaccine. Participants were also asked the criterion question of their estimate of the probability that they would receive the COVID-19 vaccine within the next 2 years.

## Study 1: Results

Prior to examining predictive relationships, the items comprising the internal and external, stable and unstable dimensions of attribution about COVID-19, and repression and sensitization about COVID-19 were subjected to a principal components analysis. Eight components with eigenvalues greater than one were noted, but the last two were small and uninterpretable. A six-factor solution proved to be more coherent, and the extracted components were then tested for reliability. Unreliable items were reassigned, and four items were discarded to produce six coherent and reliable dimensions. These included Internal-Stable (ability), Internal-Unstable (effort), External-Stable (powerful forces including people and task difficulty), and External-Unstable (fate and luck) attributions for COVID-19 safety, plus repression and sensitization coping responses to COVID-19 anxiety are reported in Table 2. The reliabilities for the other assessed dimensions also are reported.

**TABLE 2:** Reliability of assessments (Study 1).

Scale	Number of items	Cronbach’s α
Coronavirus Behavioral Health Mindset	30	0.92
Credibility of Science	6	0.93
Internal/Stable (Ability) attributions for COVID-19 safety	7	0.84
Internal/Unstable (Effort) attributions of COVID-19 safety	6	0.70
External/Stable (Powerful Forces) attributions for COVID-19 safety	6	0.74
External/Unstable (Fate and Luck) attributions for COVID-19 safety	6	0.85
Repression for COVID-19 anxiety	5	0.82
Sensitization for COVID-19 anxiety	6	0.67
COVID-19 Precautions	8	0.87
COVID-19 Vaccine Receptivity	17	0.93

As noted previously in Table 1, four differences were found on demographics between the Pre-Vx and Mid-Vx samples. The Mid-Vx sample had slightly greater age, fewer minority members, greater education, and was more likely to be employed. Considering the small demographic differences between the samples, contrasts between the Pre-Vx and Mid-Vx samples were conducted with and without the covariates of age, education, white versus minority ethnicity, and education.

Because this was not a longitudinal study, the impact of the stage of the pandemic on changes in individual attitudes could not be tested directly. It was, however, possible to indirectly examine the possible impact of time by contrasting the attitudes and behaviors of the two samples who were assessed before versus after the release of the COVID-19 vaccine. Analyses of variance on the primary measures revealed significant mean differences between Pre-Vx and Mid-Vx attitudes and behaviors on five of 11 measures, as reported in Table 3, but only two of those differences remained when the impact of the demographic covariates was removed. Both Internal-Unstable (effort) and External-Stable (external forces) attributions were lower in the Mid-Vx period than the Pre-Vx period. This finding suggests that the availability of the COVID-19 vaccine was associated with a lower commitment to personal endeavor to avoid the illness, and less belief that it is a powerful challenge.

**TABLE 3:** Psychological differences between Pre-Vx and Mid-Vx Samples, with and without covariates (Study 1).

				Simple	Contrast	Covariate	Adjusted*
		Pre-Vx	Mid-Vx	*t*	*p*	*F*	*p*
CVBHM	Mean	112.86	115.40	–2.12	0.03*	0.28	0.87
	Standard deviation	16.22	17.36				
Mask-wearing	Mean	4.39	4.41	–0.57	0.57	0.02	0.90
	Standard deviation	0.84	0.88				
CV-precautions	Mean	25.89	26.14	–0.49	0.62	0.84	0.36
	Standard deviation	6.59	7.44				
Credibility of science	Mean	21.01	21.25	0.86	0.39	0.31	0.58
	Standard deviation	3.93	3.83				
Vote conservative	Mean	1.77	1.76	0.16	0.87	0.66	0.41
	Standard deviation	0.86	0.86				
Internal/Stable (Ability)	Mean	17.62	16.19	3.81	0.001**	2.11	0.15
	Standard deviation	5.22	5.14				
Internal/Unstable (Effort)	Mean	20.94	20.44	1.76	0.08	4.99	0.03*
	Standard deviation	3.83	4.03				
External/Stable (Powerful forces)	Mean	13.47	12.27	4.19	0.001***	4.79	0.03*
	Standard deviation	4.23	3.70				
External/Unstable (Fate and Luck)	Mean	86.84	85.96	2.69	0.01*	1.05	0.31
	Standard deviation	4.70	4.32				
Repression	Mean	11.25	10.64	2.07	0.04*	0.37	0.54
	Standard deviation	4.22	3.98				
Sensitization	Mean	17.57	17.59	–0.07	0.95	0.07	0.79
	Standard deviation	4.37	4.50				

***p < .0001; **p < 0.01; *p < 0.05.

The intercorrelations of the CVBHM with the other attitudinal measures are reported in Table 4. The data are presented separately for the Pre-Vx and Mid-Vx samples, but the correlations are remarkably consistent across time periods. The CVBHM, which tapped into an internal LOC for health, was positively related to Internal-Unstable attributions of causality for COVID-19, which focused on effort, and to the coping behavior of COVID-19 sensitization, or vigilance and worry, and to belief in the credibility of science. The CVBHM was negatively related to voting conservative (Republican/Libertarian), Internal-Stable (ability), External-Stable (powerful forces) and External-Unstable (fate and luck) attributions for COVID-19, and the coping behavior of repression. Credibility of science was negatively correlated with voting conservative, and the two variables showed opposite patterns of correlations with CVBHM, attributions, and coping behaviors. Internal-Unstable causal attributions for COVID-19 prevention were negatively correlated with attributions of COVID-19 prevention to Internal-Stable, External-Stable, and External-Unstable causes, while the latter three correlated positively together. COVID-19 repression and sensitization had a small inverse relation.

**TABLE 4:** Correlations among psychological variables for Pre-Vx and Mid-Vx samples (Study 1).

	CVBHM	Science credibility	Vote conserv.	Internal stable	Internal-Unstable	External-Stable	External-Unstable	Repress	Sensitiz.	Vx recept.
CVBHM	1	–0.37**	–0.48**	–0.55**	0.68**	–0.55**	–0.61**	–0.61**	0.32**	0.65**
Science credibility	0.39**	1	–0.49**	–0.47**	0.26**	–0.46**	–0.60**	–0.54**	0.15**	0.58**
Vote conservative	–0.48**	–0.46**	1	0.42**	–0.37**	0.48**	0.53**	0.46**	–0.30**	–0.54**
Internal stable (Ability)	–0.51**	–0.46**	0.38**	1	–0.20**	0.65**	0.68**	0.62**	–0.21**	–0.53**
Internal-Unstable (Effort)	0.66**	0.28**	–0.34**	–0.15**	1	–0.26**	–0.40**	–0.43**	0.34**	0.50**
External-Stable (powerful forces)	–0.58**	–0.55**	0.55**	0.64**	–0.29**	1	0.71**	0.63**	–0.15**	–0.61**
External-Unstable (fate and luck)	–0.61**	–0.62**	0.53**	0.67**	–0.37**	0.74**	1	0.73**	–0.24**	–0.64**
Repression	–0.61**	–0.55**	0.44**	0.58**	–0.41**	0.65**	0.75**	1	–0.15**	–0.59**
Sensitization	0.22**	0.07*	–0.25**	–0.18**	0.26**	–0.09*	–0.15**	–0.08*	1	0.26*

**p < 0.01; *p < 0.05. Correlations below the diagonal are Pre-Vx participants; correlations above the diagonal are Mid-Vx participants.

Correlations calculated using the demographic and psychological variables as predicators of the behavioral criteria of mask-wearing and COVID-19 prevention are reported in Table 5. In both the Pre-Vx and Mid-Vx samples, minority members were more likely than members of the white majority to wear a facemask and take careful precautions concerning COVID-19. Older individuals and better educated people in both samples also took such precautions.

**TABLE 5:** Correlations between demographic and psychological variables and COVID-related behaviors for pre-Vx and mid-Vx samples (Study 1).

	Pre-Vx		Mid-Vx		
	Mask	CV precaution	Mask	CV precaution	Vx Prob.
Gender	0.26**	0.22**	0.09	0.10	0.06
Age	–0.02	0.19**	0.10	0.34**	0.20**
White vs. Minority	0.14**	0.19**	0.11*	0.18**	–0.10
Education	0.00	0.11*	0.12*	0.21**	0.27**
Employed	–0.06	0.14**	–0.05	0.15**	0.05
CVBHM	0.60**	0.78**	0.68**	0.84**	0.53**
Vx receptivity			0.50**	0.54**	0.85**
Science credibility	0.30**	0.26**	0.28**	0.29**	0.46**
Vote conservative	–0.36**	–0.44**	–0.44**	–0.46**	–0.48**
Internal stable (Ability)	–0.26**	–0.30**	–0.39**	–0.44**	–0.41**
Internal-Unstable (Effort)	0.45**	0.59**	0.50**	0.66**	0.46**
External-Stable (Powerful forces)	–0.47**	–0.42**	–0.38**	–0.45**	–0.49**
External-Unstable (Fate and luck)	–0.48**	–0.42**	–0.47**	–0.48**	–0.51**
Repression	–0.44**	–0.43**	–0.45**	–0.50**	–0.47**
Sensitization	0.06	0.22**	0.33**	0.29**	0.23**

**p < 0.01; *p < 0.05.

The CVBHM, Internal-Unstable (effort) attributions, and belief in the credibility of science were correlated with mask wearing and other COVID-19 precautionary behavior in both the Pre-Vx and Mid-Vx samples. COVID-19 sensitization was not associated with mask-wearing in the Pre-Vx sample, but was in Mid-Vx sample, and was correlated with COVID-19 precautionary behavior in both samples. Negatively correlated with mask-wearing and COVID-19 precautionary behavior across both samples were voting conservative, Internal-Stable (ability), External-Stable (powerful forces and difficulty), and External-Unstable (fate and luck) attributions, plus the coping mechanism of COVID-19 repression.

In response to the item “I plan on taking a COVID-19 vaccine within the next 2 years,” 54.1% of Mid-Vx participants reported “definitely yes” and another 13.0% reported “probably yes,” making two-thirds favorable. Conversely, 16.3% of the participants reported themselves to be “uncertain,” 7.0% indicated “probably no” and 9.6% reported “definitely no.”

Table 5 provides further insight into those who were receptive to the vaccine versus those resistant to it. Positively related to plans to get the COVID-19 Vx were greater education, older age, Internal-Unstable (effort) attributions about COVID, belief in the credibility of science, and COVID sensitization. Negatively related to the intention to obtain the COVID-19 Vx were voting conservative, COVID repression, Internal-Stable (ability), External-Stable (powerful forces and difficulty), and External-Unstable (fate and luck) attributions.

Additional items asked about the reasons for approach versus avoidance behaviors to the vaccine. Table 6 reports that all 17 Vx receptivity attitude items were correlated with plans to get the vaccine. The strongest positive predictor was: “I will get a COVID-19 vaccination since I do not want to risk infecting others.” Another strong predictor was: “I would support my employer requiring all workers to get the COVID-19 vaccination.” By contrast, the strongest negative predictor was “I will refuse to take a COVID-19 vaccine even if scientists say it is safe.” Another negative predictor was: “I am afraid that almost any COVID-19 vaccine will be unsafe.” This suggests that the COVID-19 Vx-receptive people are relatively community-focused, whereas vaccine resistant people tended to be personally focused.

**TABLE 6:** Relation of Vx receptivity attitudes to CVBHM, science credibility to probability of taking the COVID-19 vaccine (Study 1).

	Prob. of getting a COVID-19 vaccine.	CVBHM	Science credibility
I will get a COVID-19 vaccination since I do not want to risk infecting others. +	0.86**	0.61**	0.47**
I plan on taking a COVID-19 vaccine. +	0.84**	0.58**	0.46**
I am highly motivated to get a COVID-19 vaccine when it is available.	0.80**	0.59**	0.48**
I am confident that the new COVID-19 vaccine will be effective in preventing illness. +	0.67**	0.52**	0.43**
I will refuse to take a COVID-19 vaccine even if scientists say it is safe. +	–0.74**	–0.54**	–0.50**
I will not take any COVID-19 vaccine until it is proven to be almost 100% safe.	–0.72**	–0.36**	–0.35**
I will not get a COVID-19 vaccine since the odds of getting the virus are very low.	–0.67**	–0.49**	–0.43**
I am in good health, so I would rather take my chances with the virus than take a big risk with the COVID-19 vaccine.	–0.66**	–0.57**	–0.49**
I worry that the new COVID-19 vaccine can cause more harm than good.	–0.62**	–0.41**	–0.56**
I worry that the COVID-19 vaccine will have too many adverse side effects.	–0.62**	–0.37**	–0.57**
I would support my employer requiring all workers to get the COVID-19 vaccination.	0.62**	0.50**	0.39**
The COVID-19 vaccine is just another scam by Big Pharma to make money.	–0.61**	–0.44**	–0.49**
The COVID-19 vaccine should help control the spread of this infectious disease. +	0.59**	0.48**	0.43**
I believe that the coronavirus is not as deadly as the seasonal flu, so a vaccine is not really needed.	–0.59**	–0.60**	–0.52**
I have a positive attitude and that will protect me from COVID-19 better than any vaccine.	–0.58**	–0.36**	–0.42**
I am afraid that almost any COVID-19 vaccine will be unsafe. +	–0.49**	–0.35**	–0.48**
No person or organization should be able to require someone to take the COVID-19 vaccine.	–0.48**	–0.52**	–0.43**

**p < 0.01; + Vaccine receptivity items used in Study 2; Copyright© 2021, FifthTheory, LLC.

Structural equation modeling (SEM) was used to further clarify the relations between the demographics, CVBHM, other attitudinal and cognitive measures, and dependent variable measures of wearing a mask, taking other precautionary steps against contracting COVID-19, and intention to receive the COVID-19 Vx. It should be noted that SEM was not being used to test latent variables or demonstrate causality. Instead, SEM (LISREL 10.2, Mels, 2019) was used to clarify the relation of 15 correlated predictor variables with three correlated criterion variables. Specifically, we wished to determine if the model was stronger when the relation of the predictor variables to mask-wearing were all mediated by the CVBHM, or whether the model was stronger when the predictors were linked directly to mask-wearing, or some combination of those two extremes. The initial SEM model based on unidimensional mediation, in which all 15 independent variables predicted only the CVBHM and the CVBHM predicted the three behavioral criteria, was a poor fit [χ^2^ (42) = 193.63, *p* = 0.000001, RMSEA = 0.101]. A poor fit was also obtained by a highly saturated model in which all 15 independent variables predicted the CVBHM, and the CVBHM plus the 15 independent variables predicted each of the three criteria [χ^2^ (3) = 14.62, *p* = 0.002, RMSEA = 0.104]. The second model, however, set the stage for empirical model-trimming, in which non-significant paths were deleted as a function of the weakness of the path relationship in an iterative series of revised models. Through this process, Internal-Stable and External-Stable attributions for COVID-19 safety, repression and sensitization for coping with COVID-19 anxiety, and employment status were deleted. Although they were valid variables, they overlapped with stronger predictors of the outcome measures. The resulting final model produced an acceptable fit [χ^2^ (17) = 21.62, *p* = 0.20, RMSEA = 0.028].

**FIGURE 1:**
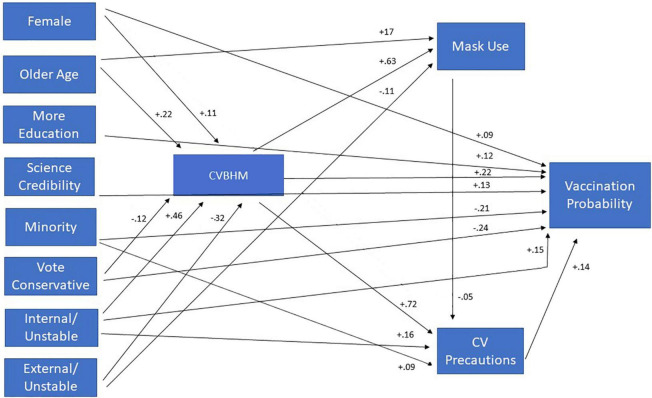
Path model between demographics, attitudes, attributions and COVID-19 safety behaviors.

Figure 1 illustrates the path model. CVBHM scores significantly predicted all three criterion measures. In addition, the structural equation model indicated that the CVBHM was positively related to Internal-Unstable (effort) attributions (β = 0.46), participant’s age (β = 0.22), and participant’s female gender (β = 0.11); and negatively related to External-Unstable (fate and luck) attributions (β = –0.32), and voting, or intending to vote, conservative in the 2020 election (β = –0.12). Mask-wearing was positively predicted by the CVBHM (β = 0.63), and negatively predicted by age (β = –0.17) and External-Unstable (fate and luck) attributions (β = –0.11). Taking other coronavirus precautions was positively related to CVBHM (β = 0.72), Internal-Unstable (effort) attributions (β = 0.16), and minority group membership (β = 0.09). Finally, the reported probability of getting the coronavirus vaccine was related positively to the CVBHM (β = 0.22), Internal-Unstable (effort) attributions (β = 0.15), and education (β = 0.12), and was negatively predicted by voting conservative (β = –0.24) and by being an ethnic minority member (β = –0.21).

Considering their relations with reluctance to get a COVID-19 vaccine, additional LISREL analyses were conducted on the psychological correlates of being an ethnic minority member and with voting conservative [χ^2^(3) = 4.25, *p* = 0.24, RMSEA = 0.034]. Minority status was positively associated with attributions of COVID-19 prevention to Internal-Unstable causes like effort (β = 0.15) and External-Unstable causes like fate and luck (β = 0.14). Minority group membership was negatively associated with voting conservative (β = –0.36), belief in the credibility of science (β = –0.19) and with education (β = –0.11). Voting conservative was associated with attributions of COVID-19 prevention to External-Stable causes like powerful others and difficulty (β = 0.31), and with attributions to External-Unstable causes like fate and luck (β = 0.14). Conservative voting also was negatively associated with belief in the credibility of science (β = –0.16), COVID-19 sensitization (β = –0.16), and with attributions to Internal-Unstable causes like effort (β = –0.12). Thus, minority members were like Conservative voters in distrusting science and attributing COVID-19 safety to fate or luck. But the two groups differed in that minority members also attributed getting or not getting COVID-19 to the Internal-Unstable causes like personal effort, whereas Conservative voters attributed getting or not getting COVID-19 to External-Stable causes like powerful other people and the difficulty of avoiding COVID-19.

## Study 1: Discussion

Study 1 demonstrated the utility of the theoretical construct of LOC and its operationalization in the CVBHM assessment in understanding individual differences in health practices related to COVID-19. The structural equation model indicated that high scorers on the CVBHM were most likely to be older individuals, females, and members of minority groups. High scorers on the CVBHM tended to make Internal-Unstable attributions for COVID-19 safety, believing that they needed to devote personal effort to defend against the virus. As a result, CVBHM scores significantly predicted important criterion measures including mask-wearing and additional preventative behaviors to avoid the virus. Low scorers on the CVBHM tended to be conservative (Republican/Libertarian) voters in the 2020 U.S. election and to believe that whether or not they contracted the virus was due to External-Unstable factors like fate and luck. Finally, the reported probability of receiving the coronavirus vaccine was positively related to CVBHM scores, Internal-Unstable (effort) attributions, and education.

Study 1 found a small number of differences in the attitudes of samples of participants surveyed before versus after the introduction of the coronavirus vaccine, after controlling for demographic variables. Both Internal-Unstable (effort) and External-Stable (powerful external forces) attributions were lower in the later Mid-Vx period than the earlier Pre-Vx period. These outcomes suggest the possibility that the release of the vaccine reduced respondents’ beliefs in the need for personal effort to guard against COVID, and reduced beliefs in the difficulty of individually responding to the pandemic or of relying on powerful political figures to offer safety.

Further analyses of Study 1 data indicated that the two demographic groups most resistant to receiving a COVID-19 vaccination (i.e., conservative voters and ethnic minority members) had similarities and differences. Both conservative voters and ethnic minority members expressed low belief in the credibility of science and endorsed attributions of COVID-19 health to External-Unstable causes like fate and luck. Voting conservative also was positively associated with attributions of COVID-19 safety to External-Stable causes like powerful others and the magnitude of the challenge. Such voting was associated negatively with attributions to Internal-Unstable causes like effort and with sensitization coping. By contrast, ethnic minority status was positively associated with attributions of COVID-19 health to Internal-Unstable causes like effort. Thus, while conservatives and minorities both believed in luck more than science, they diverged in terms of their belief in protection by personal effort versus powerful forces.

The foregoing findings suggest the value of different public health campaigns targeted toward the two groups. There is a precedent for such attitude changes in the safety LOC literature. Whereas safety LOC is a generally stable personal disposition, it can be changed (Huang and Ford, 2012). In an experimental investigation, Nykanena et al. (2019) enrolled 464 students from eight vocational schools for practical nurses, carpenters, and other trades who participated in a school-based cluster randomized, controlled intervention study conducted in Finland. The 12-h “Attitudes to Work” intervention program included topics such as identifying behavioral strategies for preventing accidents and overcoming barriers to safe work, assertive behavior in safety communication, and setting personal occupational safety and health goals. The control condition received written material on safety and participated in normal school activities and lessons. Results indicated that the intervention improved safety motivation compared to baseline and the control condition. In addition, SEM indicated that the internal safety LOC mediated the effect of the intervention on safety motivation. Also supporting the possibility of change, Dave et al. (2019) developed a new Aviation Safety LOC scale using 476 European pilots. They found that flight hours predicted internal LOC after controlling for age, indicating that work experience can enhance internal attributions of control.

Kenrick et al. (2018) demonstrated that even anti-scientific thinking was amenable to skillful interventions. Based on these precedents, and the present results, conservatives who are attuned to powerful others might respond to a campaign that states that “Donald, Ivanka, Erik, and Jared all took the COVID-19 vaccine. Make America Healthy Again!” By contrast, ethnic minority group members might respond to a campaign that states: “Deaths from COVID-19 are highest in the minority community. Get the vaccine because Black Lives Matter!” Similar custom-tailored messages could be crafted for other groups. The hope of public health officials is to get everyone on board the vaccination highway, even if it takes different on-ramps. Additional implications of these findings are discussed in the final discussion.

### Concurrent Reports on COVID-19 Prevention and Vaccination Intentions

While Study 1 was in progress, other studies appeared on COVID-related phenomena. While these findings came too late to influence the methodology used in the present research, the findings can be compared with ours. Our results concerning the LOC construct are consistent with the findings of Olagoke et al. (2021) who found that COVID-19 vaccine intentions were negatively related both to an external health LOC (*r* = –0.23) and to religiosity (*r* = –0.17). The present results concerning the credibility of science are congruent with the findings of Sanchez and Dunning (2021) who reported in several studies that positive feelings toward scientists, rather than political attitudes or knowledge, related to who was willing to engage in pandemic-reducing behaviors. In one of their studies, a warmer attitude toward scientists was related to both useful COVID-19 abatement attitudes (*r* = 0.33) and COVID-19 behaviors (*r* = 0.28). Another study in the same report found that asking participants to watch a scientist discuss hydroxychloroquine on conservative Fox News led people to greater endorsement of COVID-19 abatement behaviors. Again, those with greater warmth toward scientists were more likely to engage in favorable COVID-19 preventive behaviors (*r* = 0.31) and had more COVID-19 concern (*r* = 0.23).

While Sanchez and Dunning (2021) found that attitudes toward scientists and not political attitudes influenced COVID-19-related behaviors, our results indicated that the two variables had independent significant effects. Related results were reported by Huynh and Senger (2021) who investigated intellectual humility versus intellectual arrogance, with arrogance associated with anti-science attitudes. They found that intellectual humility was negatively related with anti-vaccination attitudes overall (*r* = –0.46) and positively related to intentions to vaccinate against COVID-19 (*r* = 0.20). Hierarchical multiple regressions revealed that intellectual arrogance predicted anti-vaccination attitudes and lower COVID-19 vaccination intentions above and beyond demographic and personal factors.

With respect to intention to take the COVID-19 vaccine, Zampetakis and Melas (2021) conducted a study in Greece. Participants were between 18 and 70 years of age (*n* = 1006) and from October 1 to November 5, 2020, engaged in an anonymous online survey. Measures of dispositional optimism (Life Orientation Test-Revised, e.g., “I am always optimistic about my future.”), faith in intuition (the Rational Experiential Inventory, e.g., “My initial impressions of people are almost always right.”), the General Risk Propensity Scale (e.g., “I am attracted, rather than scared, by risk.”), and acquiring resources mindset (e.g., “When I see something of value I go after it without much thought.”) were included as individual difference variables. Significant effects on intentions to vaccinate included male gender (*b* = –0.16), risk-taking propensity (*b* = 0.12) and an acquiring resources mindset (*b* = 0.11). Finally, Pfattheichera et al. (2021) reported that the intention to get vaccinated against COVID-19 was correlated with empathy for those most vulnerable to the virus (*r* = 0.26) and with belief in herd immunity through vaccination (*r* = 0.58).

## Study 2

In Study 1, we found that those who scored higher on the CVBHM were disposed to take personal responsibility for their personal health, were less prone to attribute their health outcomes to external forces that were outside of their control, and were prone to trust science and seek out informative news, all of which are qualities that may be prized by prospective employers who seek hard-working and dedicated employees. Our prior studies on the CVBHM reported that high scorers were more agreeable, conscientious, open to new ideas and experiences and interested in social and artistic careers (Cunningham et al., 2021), which also are desirable qualities to many employers.

The CVBHM also predicted the health behaviors of mask-wearing and receptivity to the COVID-19 vaccine, with the top predictor of the latter being “I will get a COVID-19 vaccination since I do not want to risk infecting others.” That result raised the interesting question about whether vaccine receptivity was related to social interest and sensitivity, and other desirable employee attributes. Study 2, therefore, was conducted to broaden our understanding of the psychology of COVID-19 vaccine receptivity attitudes with particular application to employment settings. Employers who seek vaccinated employees are keenly interested in whether they are screening in highly talented potential employees versus inadvertently excluding them. Thus, Study 2 was focused on the question of whether those with high vaccine receptivity also might be favorably disposed toward employment opportunities and customer service.

FifthTheory’s Customer Experience Mindset (CXM) assessment is an attitudinally based measure intended for use in customer-facing positions (Billings et al., 2020a,b). The original version of the assessment is a 14-item measure assessing three sub-facets: Customer Experience Emphasis (the extent to which a respondent believes customer experiences to be important), LOC (the extent to which a respondent believes he/she controls customer experience events and outcomes) and Emotional Intelligence (being in tune with, and able to label, customers’ feelings, as well as one’s own feelings, and have this understanding guide one’s behavior in service situations). Previous research has found the CXM assessment to be a valid and reliable predictor of service-related performance criteria. Specifically, in two studies, the measure was found to be strongly related to the ability to calm down individuals who are upset, going out of one’s way to provide service or assistance to others, and the overall ability to provide effective service or assistance (Billings et al., 2020a,b). Billings and Jones (2020) also examined CX mindset differences pre-COVID-19 pandemic and mid-pandemic. It was found that respondents scored significantly higher mid-pandemic than pre-pandemic on the CXM items related to Customer Experience Emphasis. This means that, in general, mid-pandemic respondents strongly believe that customers care about an easy enjoyable shopping experience and providing this type of experience will help retain and keep customers satisfied. The results also indicated that the mid-pandemic respondents felt they had less control over improving a customer’s experience and making the experience a positive one.

Expanding on the previous CX mindset research, the present Study 2 examined the relationship between COVID-19 vaccine receptivity, readiness to work, and CX mindset, with the expectation that Vx receptivity would be related both to the CX mindset and to the desire to return to external employment. We also measured COVID-19 risk exposure versus carefulness and emotional distress during the pandemic. Because the online survey venue limited the number of items that could be posed, the CVBHM and some of the other assessments used in Study 1 could not be included in Study 2. They will be used again in Study 3, which will extend Study 2’s focus on vaccine receptivity and work by examine people who volunteer to work to make the vaccine available to others.

## Study 2: Methods

### Participants

A national sample of 222 respondents, age 18 and older, were obtained from the Survey Monkey platform on March 21, 2021. There were somewhat more females (60.4%) than males (39.6%) in the sample. Respondents were a diverse cross-section of the U.S. More respondents came from South Atlantic states such as Georgia and Florida (21.6%), Middle Atlantic states such as New York and Pennsylvania (16.2%), Pacific states such as California (16.2%), and East North Central states such as Illinois and Indiana (15.3%), than elsewhere, but all regions were well-represented. Finally, a plurality of the respondents were in the 45–60-year-old age range (34.2%). Due to limitations in the number of questions, other demographics were not assessed.

### Measures

#### Vaccine Receptivity

The vaccine receptivity measure employed in Study 2 was a reduced version of the measure used in Study 1. It included six items measuring confidence in the vaccine and intention to receive the vaccine. It was highly correlated with the longer Study 1 measure (*r* = 0.74) and had high reliability (α = 0.92).

#### COVID-19 Exposure Risk

This three-item measure focuses on the practice of attending gatherings in which people were closer than six feet and not wearing face masks, being in areas where the coronavirus is widespread (such as a nursing home, hospital, correctional institution, food processing plant, etc.), and being in frequent contact with individuals who might have the coronavirus. In a prior investigation (*n* = 271), the internal consistency of the Exposure Risk measure was α = 0.78. In this sample, it was a bit lower but still useable, α = 0.58.

#### Emotionality

This five-item measure examines the impact of the pandemic on respondents’ feelings of distress, including feeling tense, nervous, sad, upset, and nothing being fun anymore (α = 0.83).

#### Work Readiness Attitudes

This 10-item measure is designed to assess current attitudes about motivation and readiness to work during the pandemic (Cunningham et al., 2020a,b). Example item: “After all that has happened, I appreciate the opportunity to work now more than ever.” (α = 0.75).

#### CX Mindset

A brief three-item version of the CXM scale was used (e.g., “There are many things employees can do to improve a customer’s shopping experience, including being courteous and respectful”). In the CXM validation sample (*n* = 264), the three items produced an alpha reliability of 0.62, and the correlation between the three-item scale and the original 14-item scale was *r* = 0.75. The reliability in this sample was lower (α = 0.54) but still adequate for hypothesis testing.

## Study 2: Results

Table 7 presents the correlations between vaccine receptivity and the other measures. A higher level of vaccine receptivity was related to greater work readiness, a stronger CX mindset and lower exposure risk to COVID. Greater CX mindset also was related to greater work readiness. At first consideration, it might seem paradoxical that people with higher exposure risk to COVID-19 were less receptive to the vaccine. Yet, both variables are consistent with flouting CDC recommendations. A person who takes risks by refusing to wear a mask and maintain a 6’ social distance, and by visiting people and places where COVID-19 is common also seems to be inclined to take the risk of not being vaccinated.

**TABLE 7:** Relations between vaccine receptivity and other variables (Study 2).

	Vaccine receptivity	Exposure risk	Emotional distress	Work readiness	Customer service mindset
Vaccine receptivity	1				
COVID-19 exposure risk	–0.16*	1			
Emotional distress	–0.04	0.10	1		
Work readiness	0.27**	–0.20**	–0.37**	1	
Customer service Mindset	0.34**	–0.43**	–0.26**	0.41**	1

*p < 0.05; **p < 0.01.

Vaccine receptivity was not related to emotional distress about COVID-19, but greater emotional distress was associated with lower work readiness and lower CX mindset. Thus, individuals who were receptive to the COVID-19 vaccine were not only more inclined toward biosafety, but they were more eager to work, and were disposed to be more customer-focused than individuals who were resistant to receiving a COVID-19 vaccination. Thus, it is likely that an employer who insists that new employees be vaccinated against COVID-19 will not be rejecting the best job applicants. Instead, the selection of applicants who are favorably disposed to the COVID-19 vaccine seems likely to produce a workforce of eager, hard-working, and customer-focused employees who also are committed to keeping themselves and the workplace safe.

## Study 3

A consistent finding in Study 1 and Study 2 was that the CVBHM and vaccine receptivity measures were associated with a more prosocial orientation. Those findings led us to focus Study 3 on exploring the psychology of people who were fully committed to the public health agenda of vaccination. Thus, Study 3 explored the attitudes and motives of individuals who volunteered to assist local public health planners in the delivery of COVID-19 vaccinations in an urban community. As these vaccination volunteers included healthcare as well as non-healthcare volunteers, we expected their CVBHM scores to be particularly high, and we also looked at other assessments of this unique group. Specifically, Study 3 looked at the demographics, CVBHM scores, and other attitudes, plus the self-reported motives of individuals who engaged in formal volunteering at a COVID-19 vaccination center.

## Study 3 Methods

### Participants

The Louisville Metro Public Health and Wellness Department developed a database of past and potential volunteers. LouVax Broadbent, the Louisville KY mass immunization site, used this database to recruit volunteers to assist in daily vaccination activities during the 17 weeks of operation. As an incentive, potential volunteers were promised a vaccination for themselves after a maximum of 40 h of service if they had not already obtained it. A total of 2,606 individuals provided some LouVax volunteer service. Email addresses for those volunteers were used to deliver an invitation to participate in an online survey on their attitudes and behaviors involving COVID-19. The Study 3 sample (*n* = 418, 16% of the total) responded to this request between April 12 and April 20, 2021.

Table 8 presents the demographic means and standard deviations of the Study 3 Post-Vx sample in contrast with the combined Study 1 Pre-Vx and Mid-Vx samples. It was most appropriate to contrast the Study 1 and Study 3 samples because they were both drawn from limited locations in the middle of the U.S., and those two studies contained variables that the national sample employed in Study 2 did not. The Study 3 Post-Vx sample did not differ in gender from the Pre- and Mid-Vx samples but was significantly older, more likely to be white, with more education, and higher rates of employment than the Study 1 sample. Because the Study 1 frequencies were presented previously, only the Study 3 frequencies are mentioned here. In Study 3, 27.8% of participants identified as male, 71.8% identified as female, and 0.5% reported “other” or declined to answer. As noted, Study 3 sample participants were generally older than the Pre-Vx and Mid-Vx samples. In the Study 3 Post-Vx sample, 2.6% were in the 18–24-year-old category, 30.0% ages 55–64, and 35.0% age 65 or older, and the remainder in other age categories.

**TABLE 8:** Demographics differences between Study 1 (Pre-Vx and Mid-Vx) versus Study 3 (Post-Vx) samples (Study 3).

		Pre-Vx	Mid-Vx	*t*	*p*
Variables	*N*	794	418		
Sex	Mean	1.69	1.72	–1.21	0.23
	Standard deviation	0.46	0.45		
Age	Mean	2.55	5.74	–39.76	0.0001
	Standard deviation	1.26	1.36		
Ethnicity	Mean	1.19	1.05	8.20	0.0001
	Standard deviation	0.39	0.21		
Education	Mean	3.47	6.88	–30.66	0.0001
	Standard deviation	1.86	1.76		
Employed	Mean	2.60	3.82	–16.74	0.0001
	Standard deviation	1.23	1.20		

*Sex Male = 1; Other = 2; Female = 3.*

*Age 1 = 17 years or younger; 2 = 18–24; 3 = 25–34; 4 = 35–44; 5 = 45–54; 6 = 55–64; 7 = 65–74; 8 = 75 and older.*

*Ethnicity White = 1; Minority = 2.*

*Education 1 = < HS or GED; 2 = HS or GED; 3 = 1–2 years college; 4 = Assoc. degree; 5 = 3–4 years college; 6 = Bach. degree 7 = some post-grad.; 8 = Masters; 9 = Doctoral or prof. degree.*

*Employed 1 = unemployed; 2 = part-time or student; 3 = dislocated due to COVID; 4 = retired or full disability; 5 = full-time employed.*

The Study 3 Post-Vx sample also had remarkable educational attainment, with only 2.1% reported having high school diplomas, G.E.D., or less, 28.5% had 4-year college degrees, and 49.3% had a Master’s or Doctorate degree, with the rest in other categories. It should be noted that because the program focused on the direct administration of the vaccine, many of the volunteers were nurses, pharmacists, and physicians. The Post-Vx sample had little diversity, instead being 95.4% white and 4.6% minority, including 2.2% black or African American. Less than 1% also reported themselves to be Hispanic (0.7%). In terms of employment in the Post-Vx sample, the largest proportion were retired (45.6%), while 31.7% worked full-time, 13.5% had part-time employment, and 1.2% were full-time students. Economic dislocations due to COVID-19 affected only 1.9% of the sample.

In terms of political affiliation, 16.1% reported their orientation to be Republican or Libertarian, 15.9% Independent or Other, and 68.1% Democratic. In the past election 12.9% voted for the Republican candidate Donald Trump, 82.4% voted for the Democratic candidate Joseph Biden, and 3.5% voted for third-party candidates.

### Measures

The Study 3 survey was similar to that used in Study 1 but included additional questions about people’s motive for volunteering to help with the administration of COVID-19 vaccinations. Participants were asked to report “The most important reason that motivated me to originally volunteer to help with COVID-19 vaccinations was” and were given five alternatives: “to help other people by preventing them from getting sick with COVID”; “to show that I am a compassionate and helpful person”; “to honor a family member who suffered from COVID-19 or another disease”; “to get the COVID-19 vaccine myself at the first possible opportunity”; and “Other.” A forced-choice response format was used to encourage respondents to thoughtfully identify their top motives and to discourage the tied scores that can occur with a rating scale. Reports in the “Other” category will be presented below.

To further examine personal motives, respondents were asked to report the number of extended family members who contracted COVID-19 including oneself, the number of friends or neighbors who contracted COVID-19, the number of work associates who contracted CV, and whether they knew someone who died from COVID-19. Because 99% of the Study 3 participants were vaccinated, they were asked only seven of the 17 vaccine receptivity items used in Study 1, three of which also were used in Study 2.

## Study 3: Results

Study 3 participants did not differ from the Pre-Vx and Mid-Vx participants in Study 1 in terms of the CVBHM and COVID-19 sensitization coping style, after controlling for demographic differences in gender, age, ethnicity, education, and employment. Yet as reported in Table 9, the Vx delivery volunteers were higher on COVID-19 precautions, but lower on mask-wearing. The former variable probably motivated the Post-Vx group to obtain vaccinations and the latter effect may have been caused by the sense of safety afforded by the inoculation. Note that this assessment was conducted prior to the spread of the COVID-19 Delta and Omicron variants.

**TABLE 9:** Attitude, attribution and coping differences between Study 1 (Pre-Vx and Mid-Vx) versus Study 3 (Post-Vx) samples (Study 3).

				Simple	Contrast	Covariate	Adjusted*
		Pre- and Mid- Vx	Post-Vx	*t*	*p*	*F*	*p*
CVBHM	Mean	114.09	125.94	–13.736	0.000	0.08	0.77
	Standard deviation	16.82	12.19				
Science credibility	Mean	21.12	23.80	12.735	0.000	26.20	0.0001
	Standard deviation	3.89	2.84				
Vote Conservative	Mean	1.76	1.31	9.861	0.000	21.16	0.0001
	Standard deviation	0.86	0.69				
Internal-Stable	Mean	16.94	12.53	15.939	0.000	9.70	0.002
	Standard deviation	5.23	3.93				
Internal-Unstable	Mean	20.71	22.56	-8.644	0.000	7.76	0.005
	Standard deviation	3.93	3.14				
External-Stable	Mean	12.91	9.77	16.198	0.000	6.57	0.01
	Standard deviation	4.03	2.48				
External-Unstable	Mean	86.42	83.11	14.590	0.000	12.84	0.0001
	Standard deviation	4.54	3.10				
Repression	Mean	10.96	8.11	13.654	0.000	4.72	0.03
	Standard deviation	4.12	2.88				
Sensitization	Mean	17.58	15.67	7.271	0.000	0.001	0.97
	Standard deviation	4.43	4.08				
Mask-wearing	Mean	4.41	4.15	4.772	0.000	8.29	0.004
	Standard deviation	0.86	0.93				
CV-precautions	Mean	26.01	27.54	–4.151	0.000	5.59	0.02
	Standard deviation	7.01	5.20				
Vaccine receptivity	Mean	11.69	13.61	10.93	0.000	20.92	0.0001
	Standard deviation	2.95	1.57				

*Covariates were gender, age, ethnicity, education, and employment.

Compared to the Study 1 participants, the Study 3 Vx delivery volunteers were significantly higher on belief in science credibility, lower on likelihood of voting conservative, and lower on COVID-19 repression coping style. They also were less likely to attribute COVID-19 safety to Internal-Stable qualities like personal ability, External-Stable variables like powerful entities or difficulty, and External-Unstable factors like fate and luck. Instead, the Study 3 Vx delivery volunteers were more likely to attribute COVID-19 safety to Internal-Unstable variables like personal effort, which was consistent with their COVID-19 precaution scores. Thus, the Study 3 Vx delivery volunteers demonstrated a highly convergent profile with the Study 1 participants who reported an intention to obtain the vaccine.

In terms of the Study 3 participants motives for volunteering, as Table 10 shows, most of them (59.3%) reported that their reasons were prosocial and community-focused: “to help other people by preventing them from getting sick with COVID.” About a third of the volunteers (35.1%) reported more egocentric, personally focused motives: “to get the COVID-19 vaccine myself at the first possible opportunity.” Only 3.3% admitted impression-management motives, “to show that I’m a helpful and compassionate person.” No one endorsed the desire to honor someone else who had that or another illness, although data presented below suggested that was a partial motivator. The “Other” option was selected by 2.2% participants, which revealed two additional reasons: 1.4% of participants expressed a desire to join a prosocial cause: e.g., “I wanted to support the solution”; “serve my community”; and “be part of a large public health initiative.” Another 0.8% of participants indicated a desire to meaningfully fill their time, e.g., “Wanted to have a regular schedule again, was laid off”; “had the time”; and “used the volunteer hours for work.”

**TABLE 10:** Correlations and multiple regression of demographic, attributional, and coping variables with personal versus prosocial motives for volunteering to help deliver COVID-19 Vx (Study 3).

Variable	*r*	*B*	Standard error	β	*t*	*p*
Gender	0.05					
Age	0.25**	0.06	0.02	0.18	3.18	0.002
Minority group	0.04					
Education	0.09					
Employed	0.11*	0.04	0.02	0.09	1.71	0.088
Vote conservative	0.07					
CVBHM	0.09					
CV precaution	0.02					
Science credibility	0.03					
Internal stable (Ability)	–0.01					
Internal-Unstable (Effort)	0.01					
External-Stable (Powerful forces)	–0.06					
External-Unstable (Fate and Luck)	–0.02					
Repression	–0.04					
Sensitization	–0.20**	0.13	0.05	–0.13	2.48	0.014
# of extended family members who contracted CV including oneself	–0.01					
# of friends or neighbors who contracted CV	–0.07					
# of work associates who contracted CV	0.07					
Know someone who died from CV	0.18**	–0.01	0.01	–0.11	–1.98	0.049

**p < 0.01; *p < 0.05.

A binary volunteer motive variable was created by pooling the prosocial motives of the desire to help people and to join a cause (60.7%) versus the personal motives of getting the COVID-19 vaccination oneself, conveying an image of compassion, and structuring time (39.3%). As Table 10 reports, prosocial motives for volunteering to help deliver COVID-19 vaccinations were more often reported by older individuals who were employed and by individuals who knew someone who had died of COVID-19. Personal motives for volunteering to help deliver COVID-19 vaccinations were more likely to be reported by those who employed the COVID sensitization coping style.

Although participant age was correlated with knowing someone who died from COVID-19 (*r* = 0.19) in the multiple regression analysis reported in Table 10, both age and knowing an acquaintance who died from COVID-19 were independent significant predictors of the prosocial motive. The COVID-19 sensitization coping style also remained significantly related to personal motivation, while the effect of employment was reduced to a trend.

## Study 3: Discussion

Study 3 effectively replicated Study 1 by demonstrating that those who obtained vaccination against COVID-19 themselves, and who helped others to obtain the inoculation, had high scores on the CVBHM, and attributed COVID-19 safety to personal effort, rather than individual ability, powerful forces, or luck. The Study 3 volunteers also were consistent with the Study 1 individuals who intended to get the vaccine in having a strong belief in the credibility of science, low COVID repression coping style, and low likelihood of voting conservative. The Study 3 volunteers also tended to be well-educated, older, and retired. The latter variables, plus low COVID sensitization coping style and knowing someone who died from COVID-19, were related to prosocial rather than personal motives for volunteering.

While Study 3 was being conducted and analyzed, other research was reported on the characteristics of COVID-19 vaccine volunteers. These findings came too late to influence the methodology used in the present research, but the findings can be compared with ours. Mak and Fancourt (2021) analyzed data from 31,890 adults in the United Kingdom to understand those who volunteered to provide services to individuals impacted by the pandemic. Their study commenced on March 21, 2020, the same date as ours, and involved online weekly data collection from a heterogeneous sample. A variety of individual differences were assessed and three types of volunteering during the pandemic were identified: formal volunteering, social action volunteering, and neighborhood volunteering. Regression analysis showed that the pattern of voluntary work was related to demographic background, socio-economic, personality, and psychosocial factors. For example, formal volunteering was predicted by higher education. Social action volunteering was associated with higher education, female gender, being currently employed, and higher income. Neighborhood volunteering was more common in females and with increasing age. In terms of personality, those more likely to volunteer had higher scores on extraversion, openness, and agreeableness. Higher rates of social action volunteering also were associated with neuroticism. Neighborhood volunteering was related to conscientiousness. Those with high levels of social support and a large social network displayed a higher rate of volunteerism overall, while those diagnosed with mental health conditions had 32% higher odds of engaging in formal volunteering. LouVax volunteers engaged in what Mak and Fancourt’s (2021) study would call formal volunteering, and the two studies converged in finding that higher education was associated with such volunteering. The LouVax volunteers also were like Mak and Fancourt’s social action volunteers in being female and employed and with their neighborhood volunteers in being in the upper age categories.

Our finding that the primary motive for the LouVax volunteers was prosocial also was consistent with Wakefield and Khauser’s (2021) small-scale (*n* = 130) study of British respondents. They reported that people’s strength of identification with their local community positively predicted their willingness to engage in community-related prosocial normative behavior, in the form of obtaining vaccination.

## General Discussion

To recapitulate, Study 1 found relations between the CVBHM, belief in the credibility of science, voting conservative, and attributions for COVID-19 safety to effort with the wearing of masks and other biosafety precautions, including receptivity to the COVID-19 vaccine. Study 2 carried those findings further by examining the relation of vaccine receptivity to work readiness and customer experience mindset and COVID-19 exposure risk. Study 2 suggested that those inclined to seek out a COVID-19 vaccination or comply with a vaccine mandate are likely to be eager employees, more customer-focused associates, and be lower in COVID-19 exposure risk than vaccine resistors. Study 3 examined a unique population who chose to volunteer to help distribute the COVID-19 vaccine for either prosocial or personal motives. The majority (60.7%) expressed prosocial motivation and they also demonstrated high CVBHM scores, greater willingness to attribute biosafety to their individual effort, lower COVID sensitization coping style, greater age, greater likelihood of being employed or retired rather than unemployed or in school, and more likely to have known someone lost to the pandemic.

**FIGURE 2:**
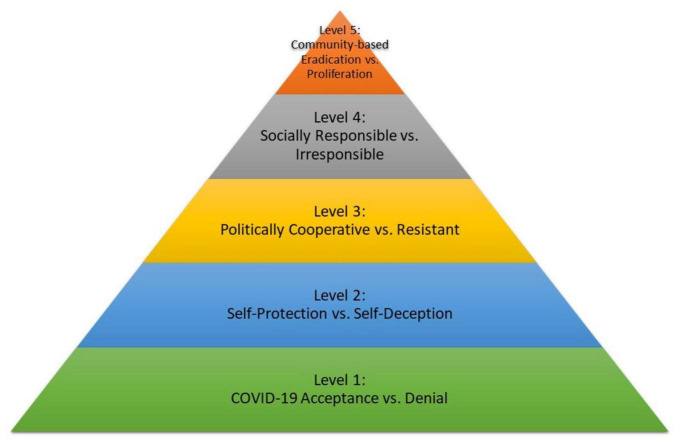
COVID-19 mindset hierarchy.

In reflecting on these empirical outcomes, an *a posteriori* model of COVID-19 behavioral health emerged that we call the COVID-19 Mindset Hierarchy. The model is intended to convey five increasing levels of psychological maturity in addressing the coronavirus and those levels are intended to parallel Maslow’s (1954) Hierarchy of Needs, Erikson’s (1963) stages of psychosocial development, Kohlberg’s (1984) stages of moral development, and Gilligan’s (1977) developmental model of caring. The COVID-19 Mindset Hierarchy (see Figure 2) suggests that individuals can be classified from least to most psychologically mature with respect to COVID-19 as a function of their focus on avoidance, self-protection, ideological resistance, social responsibility, or community protection:

### Level 1: COVID-19 Acceptance Versus Denial

Research documented that denial of the reality of COVID-19 predicted by repression and attributions of COVID-19 safety to personal ability or fate or luck, was expressed as infrequent handwashing and lack of other COVID-19 precautions. Frequent handwashing, social distancing, and wearing protective gear reflect acceptance of, and realistic responses to, the pandemic.

### Level 2: Self-Protection Versus Self-Deception

Findings revealed self-protective COVID-19 precautions, predicted by sensitization, belief in the credibility of science, and attributions of COVID-19 safety to personal effort, were expressed as intention to obtain the vaccine, a self-protective step. Self-deceptive responses included deliberate avoidance of the vaccine in favor of religious practices or unscientific remedies such as hydroxychloroquine and Ivermectin, which could not only be ineffective against COVID-19, but self-destructive.

### Level 3: Politically Cooperative Versus Resistant

The politicization of responses to the pandemic should have been scrupulously avoided, but the sad fact is that it is now ideologically linked in the U.S. While the majority of Americans cooperate with safety and vaccination mandates issued by the CDC and many state governors, a significant minority are resistant. Politically based resistance to COVID-19 safety measures, predicted by voting conservative and attributions of COVID-19 safety to powerful others, was expressed as resistance in the form of public objections to wearing a mask or obtaining the vaccine.

### Level 4: Socially Responsible Versus Irresponsible

Socially responsible COVID-19 abatement, predicted by CVBHM and CX Mindset, was expressed as consistent mask-wearing and other COVID-19 precautions even after receiving the vaccine. Socially irresponsible actions include knowingly exposing others to the virus after a positive COVID-19 test result and even weaponizing the illness in settings like schools or retail establishments by shaking unwashed hands or intentionally coughing on others.

### Level 5: Community-Based Eradication Versus Proliferation

Results revealed that community-committed COVID-19 elimination, expressed by self-reported motives to help others to avoid illness, was expressed as volunteering to deliver COVID-19 vaccines. This Level 5 state is needed to eradicate the pandemic globally and is relevant in terms of the number of citizens in each country who get vaccinated. Helping poorer countries develop and distribute the vaccine, especially as new variants evolve, is highly reflective of Level 5 actions attempting to prevent the proliferation of the pandemic. It is recognized that each level of response to COVID-19 involves political implications not just our Level 3. In addition, responses to the pandemic that are correlated with a conservative political orientation are ranked as less psychologically mature than responses correlated with a progressive political orientation. That was also the case with the developmental models on which the COVID-19 Mindset Hierarchy was based (Fishkin et al., 1973) and with analyses of the motivational underpinnings of conservatism (Jost et al., 2003). It is important to recognize that the three studies presented here produced results that inspired the COVID-19 Mindset Hierarchy model, but because the studies and measures were not designed to formally establish this developmental sequence, confirmation must await additional research. Nonetheless, this hierarchical model could be useful in reminding researchers, healthcare providers, politicians and citizens of the psychological and social dimensions of COVID-19 eradication.

### Limitations

Study 1 was based on a college and university sample, so they tended to be younger and better educated than average. Study 2 was a national online sample and tended to be middle-aged; the limitations of the online context did not allow gaining a great deal of background information about them, including the CVBHM. The Study 3 sample tended to be older and either employed or retired. None of the studies used a random sample of the population, but the variety of sampling approaches and mean ages served to insure some diversity among the respondents. Thus, despite the convenience sampling methodology, the relationship of the CVBHM with internal LOC, adaptive coping with emotion, eagerness to work and to attend to customer’s needs, trust in science, willingness to take the COVID-19 vaccine, and to volunteer to help others become inoculated seem like reliable findings. It is also important to remember that research that tests correlations and patterns among variables cannot be applied directly to individuals. In other words, we expect that individual decisions about behavioral health are much more nuanced than group relations can reveal. Research using other approaches, such as qualitative studies, may be used to detect subtle differences in reasoning for behavior that may be important in designing interventions and in predicting individual attitudes and actions. Finally, refinement of the item wording and reduction in the length of the assessments used in these studies should continue. The format of items, such as our use of a forced-choice format for assessing motive for volunteering to help with COVID-19 vaccinations in Study 3, should be replicated using questions posed in different ways to verify the validity of the present results.

### Future Directions

Khalifeh et al. (2021) recently suggested the relevance of collectivism and individualism to COVID-related behaviors. Individuals with a collectivistic orientation tend to define themselves based on their group affiliations, aim to align with the goals of their respective groups, and give priority to these group goals. Those with individualistic orientations tend to focus on their self-concept separately from their groups, have personal goals that may not overlap with the goals of their respective groups, and tend to prioritize personal goals over the group’s goals. Between April and September of 2020, those investigators gathered data from 433 Michigan undergraduates (73% female; 84% White). They found that collectivism was correlated with a composite measure of COVID-19 related worry (*r* = 0.19), including worry about becoming infected, family members getting infected, and about infecting others. Collectivism also was correlated with a composite measure of preventative actions (*r* = 0.14) including social distancing, hand washing, and self-isolating. Individualism was not correlated with either measure. In addition, using an experimental approach, Courtney et al. (2021) reported that when collectivism was primed, individuals responded to a COVID-19-based mortality reminder with a significant increase in health intentions, including social distancing and mask wearing. In a second study, when mortality was made salient, priming individualism led to reduced vaccination intention compared to collectivism. Based on the COVID-19 Mindset Hierarchy model, it is reasonable to expect that activating collectivism and mortality will be related to the CVBHM, CX mindset and volunteering to help distribute the COVID-19 vaccine, but verification will require additional research.

In the late fall and winter of 2021–2022, the coronavirus vaccination rate in the U.S. continued to increase and the death rate declined, only to be followed by the potentially dangerous COVID-19 Omicron variant, causing a new surge of cases. Because the COVID-19 vaccine is not equally available across the world, and it remains to be seen whether similar relations between the CVBHM and the COVID-19 Mindset Hierarchy model to biosafety responses will be found in countries with different political dynamics than the U.S. Nonetheless, we hope that the instruments used in this study are employed in other countries, to allow comparison of results.

When the pandemic finally winds down or the virus becomes endemic, individuals will still need to adjust to their losses, both of people and of a sense of safety and stability. We have informally observed a post-pandemic fragility among some people. It is our belief that many of the same measures that predicated responses to the coronavirus, including internal LOC and attribution of biosafety to effort, may prove to be related to adjustment to the post-pandemic world characterized by another global existential threat, that of catastrophic climate change.

## Data Availability Statement

The datasets presented in this article are not readily available because items and scales are copyrighted and the data are proprietary. Additional analyses may be obtained from the corresponding author. Requests to access the datasets should be directed to MC, michael.cunningham@louisville.edu.

## Ethics Statement

The studies involving human participants were reviewed and approved by Institutional Review Board, Human Subjects Protection Program, University of Louisville. The participants provided their written informed consent to participate in this study or clicked “continue” to signify their consent.

## Author Contributions

MC was responsible for planning, conceptualization, item generation, obtaining IRB approval at the University of Louisville, statistical analysis, reporting and editing of all studies. PD contributed to conceptualization, generation of items, was responsible for the IRB at York College, data collection in Study 1, editing of all three studies, and modification into Frontier template. ML contributed to the literature review and introduction to Studies 1 and 3, obtaining access the sample for Study 3 and the editing of Study 3. BD was responsible for online data collection for Studies 1 and 2, contributed to item generation for Study 2, the initial analysis of Study 2 and the editing of all studies. AB contributed to data analysis in Study 1, the creation of Figure 1 and the editing of all studies. RC secured the sample for Study 3, arranged the online deployment of the survey items, and provided the description of that sample. SB contributed to the conceptualization and analysis of Study 2 and the editing of all studies. JJ contributed to item generation for all three studies and provided high-level strategic feedback on the manuscript, including inspiration for the integrative model. All authors contributed to the article and approved the submitted version.

## Conflict of Interest

BD, SB, and JJ were employed by FifthTheory, LLC. RC was employed by the University of Louisville at the time this study was conducted and analyzed. The remaining authors declare that the research was conducted in the absence of any commercial or financial relationships that could be construed as a potential conflict of interest.

## Publisher’s Note

All claims expressed in this article are solely those of the authors and do not necessarily represent those of their affiliated organizations, or those of the publisher, the editors and the reviewers. Any product that may be evaluated in this article, or claim that may be made by its manufacturer, is not guaranteed or endorsed by the publisher.
